# The prevalence of female sexual dysfunction among migraine patients

**Published:** 2015-01-05

**Authors:** Mohammad Abdollahi, Mansoureh Toghae, Firoozeh Raisi, Elaheh Saffari

**Affiliations:** 1 Iranian Center of Neurological Research AND Department of Neurology, School of Medicine, Sina Hospital, Tehran University of Medical Sciences, Tehran, Iran; 2 Psychiatric and Clinical Psychology Research Center, Roozbeh Psychiatric Hospital, School of Medicine, Tehran University of Medical Sciences, Tehran, Iran

**Keywords:** Migraine, Female Sexual Dysfunction, Female Sexual Function Index Score

## Abstract

**Background: **Female sexual dysfunction (FSD) defines as any disorder in the process of sexual contact including 6 main domains, desire, arousal, lubrication, orgasm, orgasm satisfaction and pain. This study was conducted to evaluate prevalence of sexual dysfunction disorder in women with migraine headache and also find the associated factors related to migraine characteristics.

**Methods:** A total of 69 eligible woman patients fulfilling criteria for migraine participated in this study. The Female Sexual Function Index (FSFI), a multi-dimensional self-report implement for appraisal of Female Sexual Function during the past month were utilized in this study. The information related to migraine including frequency, duration of headache attack, severity of headache according to visual analog scale (VAS) score and headache impact test (HIT) score were obtained using a self-administrated questionnaire.

**Results: **About 68.4% of patients had an FSFI score < 28. In domains of desire 73.7%, arousal 64.9%, lubrication 21.1%, orgasm 33.3%, satisfaction 17.5%, and pain 40.4% of patients reported some degree of dysfunction. Among variables related to migraine characteristics, only a significant association between frequency and sexual dysfunction were recorded (P < 0.05).

**Conclusion: **FSD is prevalent among migraine patients. The frequency of a migraine attack is associated with FSD. Serotonin mechanisms such as 5HT2, 5HT3 agonist have been hypothesized as a shared etiology for migraine and sexual dysfunction.

## Introduction

Female sexual dysfunction (FSD) defines as any disorder in the process of sexual contact, including 6 main domains, desire, arousal, lubrication, orgasm, orgasm satisfaction and pain, which cause female distress and impact their relationships with partner and their quality of life.^1^^,^^2^

The prevalence of FSD varies in a range of 43-90% in studies due to different definition, studies protocol, cultural issues, environmental factors and genetics variances.^3^^,^^4^ Previous findings demonstrate that FSD is multifactorial, and there is a genetic susceptibility for sexual dysfunction that is influenced remarkably by environmental factors. Both genetic and environmental factors are involved in all dimensions of sexual function.^1^^,^^5^ In recent years, Studies discuss about the significant impact of chronic pain on female sexual function. Chronic illness and chronic pain result in less sexual satisfaction and cause some degree of sexual dysfunction.^6^^,^^7^

Primary headache especially migraine is a common cause of chronic pain and temporary disability.^8^ The prevalence of migraine and chronic headache in women is respectively 17.1% and 4%.^9^^-^^10^ Gal Ifergane in his investigation on a student sample showed that a migraine suffers have a higher sexual pain, and satisfaction disorder compared with control subjects.^11^

Bestepe et al. assessed sexual function among headache suffers using Arizona sexual experiences scale (ASEX score) and concluded that migraineurs have more difficulties with vaginal lubrication and achieving orgasm in comparison to normal samples.^12^ In a large population based study in United States, the frequency and quality of sexual relationships were affected in 86% of migraine suffer, and resulted in divorce in 26% of cases.^13^

This study is conducted to evaluate the prevalence of sexual dysfunction disorder in women with migraine headache in Iran.

## Materials and Methods

We designed a cross-sectional study to assess sexual function in women under treatment and follow-up for migraine. Our aim was to investigate the prevalence of female sexual function disorder among migraineur patients and also to identify the associated factors of FSD including headaches characteristics.

The study was conducted between April and June 2013 in a headache clinic center.

The Ethics Committee of the Tehran University of Medical Sciences approved the study. Informed consent was obtained from the patients.

Eighty-eight women with complain of headache consecutively were interviewed to participate in this study. The patients enrolled in this study met the International Classification of Headache Disorders criteria for migraine and had a sexual partner for a minimum period of last 1 year**.** 69 eligible patients were recruited.

A detailed history of headache attacks characteristics were obtained in an interview of an expert neurologist with patients. Our interview includes questions for disease duration, severity and frequency of headache attacks and attack duration. The severity of headache attacks was estimated based on visual analog scale (VAS) score. The impact of headache on quality of life was evaluated with headache impact test (HIT) score questionnaire. The Female Sexual Function Index (FSFI), a multidimensional self-report implement for appraisal of Female Sexual Function during the past month, were utilized in this study. This questionnaire consist of 19 questions in six main domains of sexual function including sexual desire, sexual arousal, vaginal lubrication, ability to achieve orgasm, orgasm satisfaction and pain and rated on a 5 points scale and full score range from 2 to 36. The cut-off point for the scale was found to be 28 in Iranian translated draft of FSFI questionnaires (sensitivity = 83% and specificity = 82%).^14^ Details about subscale cut-off point is presented in table 1.

SPSS software (version 18, SPSS Inc., Chicago, IL, USA) were applied to describe the sexual function status of subjects. Independent t-test was administrated to compare HIT Score, frequency and VAS Score in two subgroups of migraineurs regarding patients with or without FSD. P ≤ 0.05 was considered to be significant.

**Table 1:** Female sexual function and subscale scoring and cut-off points

	**Domain**	**Questions**	**Factor**	**Cut-off point**
FSFI	Desire	1, 2	0.6	3.3
	Arousal	3, 4, 5, 6	0.3	3.4
	Lubrication	7, 8, 9, 10	0.3	3.7
	Orgasm	11, 12, 13	0.4	3.4
	Satisfaction	14, 15, 16	0.4	3.8
	Pain	17, 18, 19	0.4	3.8
	Total			28

FSFI: Female sexual function index

## Results

**Figure 1:**
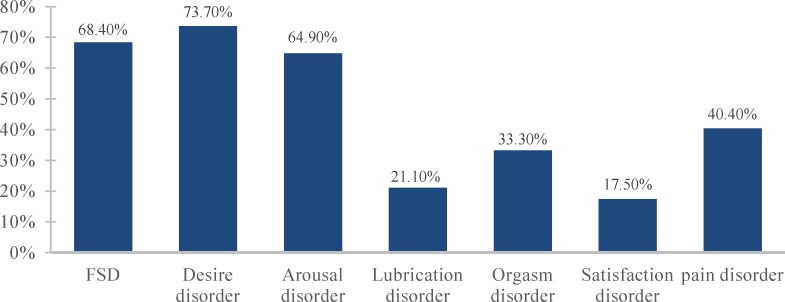
Prevalence of subscales of sexual dysfunction in female migraine patients FSD: Female sexual dysfunction

Of the 69 migraineur women who were interviewed, 12 (17%) were excluded from the analysis because of significant missing data. Average age of the migraine patients in the analysis sample was 38.11 ± 9 years (range 15-57). About 68.4% of migraine patients reported FSF score under the validated cut-off (< 28). Most common sexual dysfunction in migraine patients were observed in domains of desire and arousal (73.7 and 64.9%, respectively) (Figure 1).

**Table 2:** Comparison of variable between two subgroups of migraine patients regarding with or without female sexual dysfunction (FSD)

**Variables**	**Normal sexual function group**	**Sexual dysfunction group**	**P**
Age (year)	6.52 ± 32.73	14.65 ± 36.44	0.42
Disease (year)	4.71 ± 8.55	10.37 ± 11.6	0.18
Pain intensity (VAS score)	1.85 ± 7.36	2.21 ± 7.57	0.78
30-day headache frequency	3.46 ± 4.27	7.92 ± 8.20	0.04
HIT score	5.98 ± 62.73	18.87 ± 57.62	0.38
Attack duration (%)			0.94
> 12 h	40	38.9	
< 12 h	60	61.1	

VAS: Visual analog scale; HIT: Headache impact test

Based on our analysis of subgroups of migraine patients, a positive association was revealed between FSFI score and headache frequency (P = 0.04).

No significant association was detected between FSD and age, VAS score, migraine duration years and hit score (P > 0.05) (Table 2).

## Discussion

FSD is an important issue for many of women that disturb their emotional relationship with their partner and also impair their quality of life. Especially in migraine patients is very important to study the prevalence of FSD and assess the factors that increase its probabilities. A general population-based study in Iran estimated FSD in 31.5% of subjects^15^ obviously FSD was meaningfully more common in our migraine patients compared to general population but less common than result of Nappi et al. study in migraine and tension patients(68.4 vs. 90%).^4^ This substantial differences may reflect medical, psychological factors, socioeconomic, cultural and the characteristic of samples. Desire and Arousal disorder were the most common subscale of sexual dysfunction in migraine patients (73.7 and 64.9% respectively). In agreement with our findings, other studies in general population have reported that desire is the most common impaired domain of sexual dysfunction.^15^^,^^16^ Mostly the chronic pain disorders can adversely influence sexual desire and activity over the time.^6^^,^^7^ Gal Ifergane believed that avoidance, fear of sex and as a result lower satisfaction are associated with higher levels of sex pain disorder.^11^ The comorbid disorders especially depression can disturb neuroendocrine balance, which have some roll in sexual derive and satisfaction.^17^

Some data explain the possible role of serotonin receptors in migraine and sexual dysfunction. A shared mechanism which can suggest these association are role of 5-HT2 and 5-HT3.^18^^-^^24^ Some trials showed that anti receptors of 5-HT2 and 5-HT3 such as mirtazapine can be recommended as therapeutic agents for sexual dysfunction.^25^^,^^26^ To our knowledge there are not sufficient studies that examined the association of headache characteristics and sexual dysfunction. Consistent with our findings Bestepe et al. found no significant association between headache duration, severity and sexual dysfunction.^12^ However, dissimilar to findings of Bestepe et al.^12^ and Maizels and Burchette^27^ our data analysis detected a significant association between frequency of a migraine attack and sexual dysfunction.

The result of this study invite other researcher to conduct such studies with more sample size and more precise design and also offer a background of this issue to physician and help them to have more comprehensive attitude while interviewing the patients with complaint of migraine in clinic.

## Notes

**How to cite this article: **Abdollahi M, Toghae M, Raisi F, Saffari E. The prevalence of female sexual dysfunction among migraine patients. Iran J Neurol 2015; 14(1): 8-11.
